# Characteristics and complications of uveitis in patients referred to rheumatology: a single-center study

**DOI:** 10.55730/1300-0144.6076

**Published:** 2025-08-19

**Authors:** Handan YARKAN TUĞSAL, Serdar SEZER, Gözde ORMAN, Gülten SUNGUR

**Affiliations:** 1 Division of Rheumatology, Department of Internal Medicine, Ankara Training and Research Hospital, Ankara, Turkiye; 2 Division of Rheumatology, Department of Internal Medicine, Faculty of Medicine, Ankara University, Ankara, Turkiye; 3 Department of Ophthalmology, Ankara Training and Research Hospital, Ankara, Turkiye

**Keywords:** Uveitis, rheumatology, complications

## Abstract

**Background/aim:**

To evaluate the clinical, demographic, and complication data of patients with new-onset uveitis at the ophthalmology and rheumatology departments of a single center.

**Materials and methods:**

This retrospective study included patients newly diagnosed with uveitis who were referred to the rheumatology department for etiological evaluation between August 2021 and August 2024. Patients with a history of rheumatologic diseases associated with uveitis were not included in the study.

**Results:**

A total of 91 patients (female: n = 59, 65%) who met the inclusion criteria were enrolled in the study. The mean age at uveitis diagnosis was 45.7 ± 14.8 years. The most common form of uveitis was anterior uveitis (81.3%), followed by intermediate uveitis (14.3%) and panuveitis (4.4%). About one-third of the patients had a rheumatologic etiology (n = 29, 31.9%), with spondyloarthritis being the most common. Approximately one in five patients with newly diagnosed uveitis of rheumatologic etiology developed ocular complications (n = 5, 17.2%), 80% (n = 4) of whom had anterior uveitis and 20% (n = 1) of whom had panuveitis. Macular edema was the most common ocular complication.

**Conclusion:**

Anterior uveitis is generally regarded as more benign, but it still carries a risk of complications. Timely identification and management of systemic autoimmune diseases associated with uveitis may provide a valuable opportunity to prevent ocular complications and visual impairment.

## 1. Introduction

Uveitis, defined as inflammation of the uveal structures of the eye, is a leading cause of preventable visual loss. In Western countries, uveitis accounts for 10%–15% of total blindness, and its prevalence ranges from 12.4 to 580 per 100,000 across different geographic regions worldwide [1, 2]. Visual morbidity and the peak incidence in the 20–50-year age group represent significant socioeconomic challenges.

Etiologically, uveitis is classified into two types: infectious and noninfectious uveitis (NIU). In NIU, inflammation is primarily driven by systemic or localized autoimmune mechanisms affecting the uveal tract. In developed countries, NIU accounts for 67%–90% of uveitis cases [2]. The most common autoimmune diseases in the etiology of uveitis include HLA-B27-associated anterior uveitis (AU), spondyloarthritis (SpA), Behçet’s disease, and sarcoidosis [3]. However, systemic lupus erythematosus, rheumatoid arthritis, polychondritis, ANCA-associated vasculitis, Sjögren’s syndrome, and Takayasu arteritis (TAK) are less common etiologies.

Effective interdisciplinary collaboration is essential for the diagnosis, etiological evaluation, and long-term management of uveitis. In 2018, an international consensus was published to establish standardized referral criteria between rheumatologists and ophthalmologists for patients with ocular involvement of rheumatic diseases [4]. This initiative underscored the importance of identifying specific clinical features—such as chronic low back pain lasting more than 3 months, family history of psoriasis or ankylosing spondylitis, and personal history of SpA, inflammatory bowel disease, sarcoidosis, or Behçet’s disease—that should prompt referral to rheumatology. While the diagnostic spectrum of uveitis cases referred to rheumatology has been investigated [5–10], and pediatric uveitis complications have been well documented [11–14], data on ocular complications in adult patients referred from ophthalmology to rheumatology remain limited [15]. To the best of our knowledge, no studies in Türkiye have specifically addressed the complications observed in adult uveitis patients referred to rheumatology. This gap highlights the need for further investigation to better characterize these cases and guide appropriate multidisciplinary management.

Uveitis is classified according to the primary anatomic site of inflammation in the eye as anterior, intermediate, posterior, or panuveitis. This classification is essential for guiding etiological workup and for anticipating potential ocular complications such as macular edema, posterior synechiae, cataract, and glaucoma. AU is the most prevalent form of NIU, accounting for 47.5%–93% of all NIU cases. Intermediate uveitis (IU) is the least common form, accounting for 10%–15% of cases. Posterior uveitis and panuveitis each have a similar incidence, affecting approximately 20% of cases [2, 3]. Ocular complications can occur in all types of uveitis.

To the best of our knowledge, this is the first retrospective study from Türkiye to comprehensively report ocular complications in newly diagnosed adult uveitis patients referred to rheumatology for systemic evaluation.

## 2. Materials and methods

### 2.1. Patients and data collection

In this study, records of patients diagnosed with uveitis in the ophthalmology department and referred to the rheumatology department for etiological evaluation between August 2021 and August 2024 were reviewed. The inclusion criteria were a diagnosis of uveitis during the study period, a minimum of 6 months of follow-up, and completion of the etiological evaluation. Patients with a preexisting diagnosis of an autoimmune disease associated with uveitis, as well as those diagnosed with uveitis prior to the study period, were excluded. This approach aimed to ensure a homogeneous study population, as treatments for the underlying rheumatologic condition could independently influence the risk of complications.

Demographic, clinical, and laboratory data were obtained retrospectively from the medical records of the rheumatology department. Data on uveitis characteristics, treatment modalities, and related complications were obtained from the uveitis clinic within the same center. Results of etiological evaluations were retrieved from both the rheumatology and ophthalmology departments. Data on underlying systemic diseases, extraocular manifestations, comorbidities, and admission laboratory parameters—including erythrocyte sedimentation rate, C-reactive protein, antinuclear antibody, antineutrophil cytoplasmic antibody, human leukocyte antigen B27 (HLA-B27), and HLA-B51—were recorded. HLA-B27 testing was performed in patients with AU who did not meet the classification criteria for SpA and had no identifiable etiology. Likewise, HLA-B51 testing was conducted in patients who did not meet the diagnostic criteria for Behçet’s disease.

All ophthalmologic assessments were performed by two ophthalmologists (G.S. and G.O.) specialized in uveitis at the uveitis clinic. Standardized examination protocols were employed throughout the evaluation process. Uveitis cases were classified according to anatomic location, granulomatous status, laterality, and underlying etiology. The anatomical location of the uveitis was categorized using the International Uveitis Working Group criteria [16].

The diagnosis of SpA was established using the Assessment of SpondyloArthritis International Society (ASAS) classification criteria [17]. The International Study Group (ISG) diagnostic criteria were applied to diagnose Behçet’s disease [18]. The American College of Rheumatology (ACR)/European Alliance of Associations for Rheumatology (EULAR) classification criteria for primary Sjögren’s syndrome (pSS) and the 2022 ACR/EULAR criteria for TAK were applied to classify patients with pSS and TAK, respectively [19, 20].

The study was conducted in accordance with the Declaration of Helsinki and relevant local laws and regulations. Ethics approval was obtained from the Ankara Training and Research Hospital Clinical Trials Ethics Committee (date/no: 04 December 2024; 24–325).

### 2.2. Statistical analysis

Statistical analyses were performed using SPSS software, version 27 (IBM Corp., Armonk, NY, USA). The variables were assessed using visual methods (histograms, probability plots) and analytical tests (Kolmogorov–Smirnov and Shapiro–Wilk tests) to determine whether they were normally distributed. Descriptive statistics were reported as means ± standard deviations for normally distributed variables, medians with ranges or interquartile ranges for nonnormally distributed or ordinal variables, and frequencies for categorical variables. For comparisons between groups, Student’s t-test was used for normally distributed variables, the Mann–Whitney U test for nonnormally distributed and ordinal variables, and the chi-square or Fisher’s exact test for categorical variables. A p value < 0.05 was considered statistically significant.

Patients with missing data, including unperformed HLA-B27 or HLA-B51 tests, were retained in the overall analysis but excluded from analyses involving those specific variables. No imputation methods were applied; analyses related to HLA status were conducted using available-case analysis.

## 3. Results

### 3.1. Demographic, laboratory, and underlying systemic disease data

**Figure 1:**
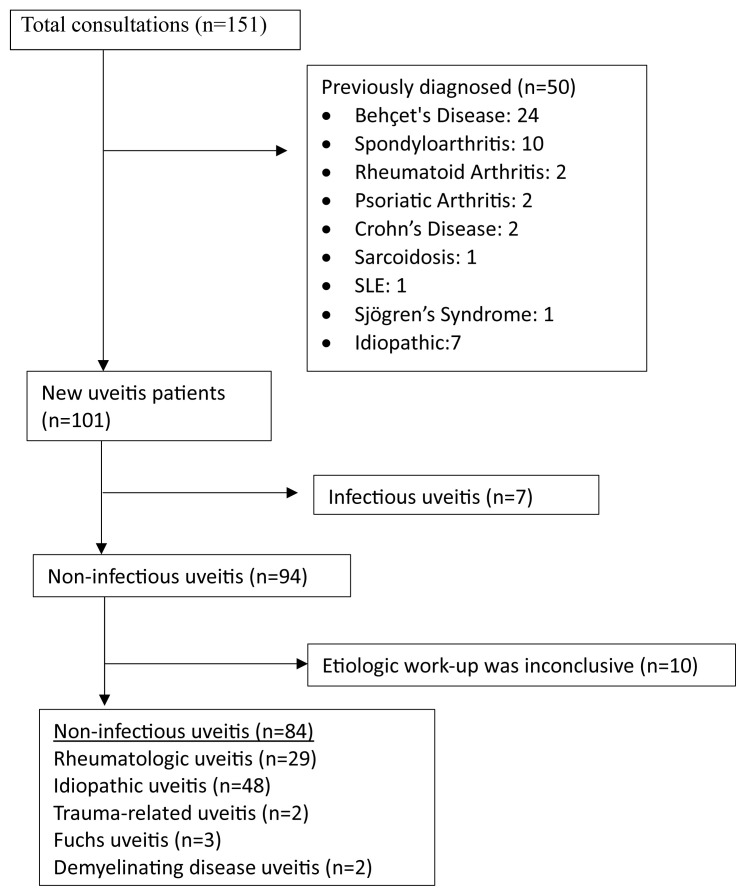
Flowchart of patient selection.

A total of 141 patients referred for uveitis evaluation were reviewed, of whom 50 were excluded due to a preexisting rheumatologic disease associated with uveitis or a diagnosis of uveitis made prior to the study period. Figure 1 depicts the flowchart for patient selection. The final cohort comprised 91 patients with new-onset uveitis, with a mean age of 45.7 ± 14.8 years; 64.8% (n = 59) were female. Among patients with NIU, 65.5% were female. The distribution of unilateral and bilateral uveitis was nearly equal. Based on anatomical classification, AU was the most common (74 patients, 81.3%), followed by IU in 13 patients (14.3%) and panuveitis in four patients (4.4%). Nongranulomatous uveitis was more common than granulomatous uveitis (78% versus 22%). A systemic autoimmune diagnosis was established in approximately one-third of the patients, with SpA being the most frequent. Infectious causes were the most frequent among nonrheumatologic etiologies. Nearly one-fifth of the patients developed complications, the most common being macular edema. HLA-B27 positivity was observed in approximately 50% of the tested patients. Detailed demographic and clinical data are presented in Table 1.

### 3.2. Noninfectious causes of uveitis according to anatomic localization

**Figure 2:**
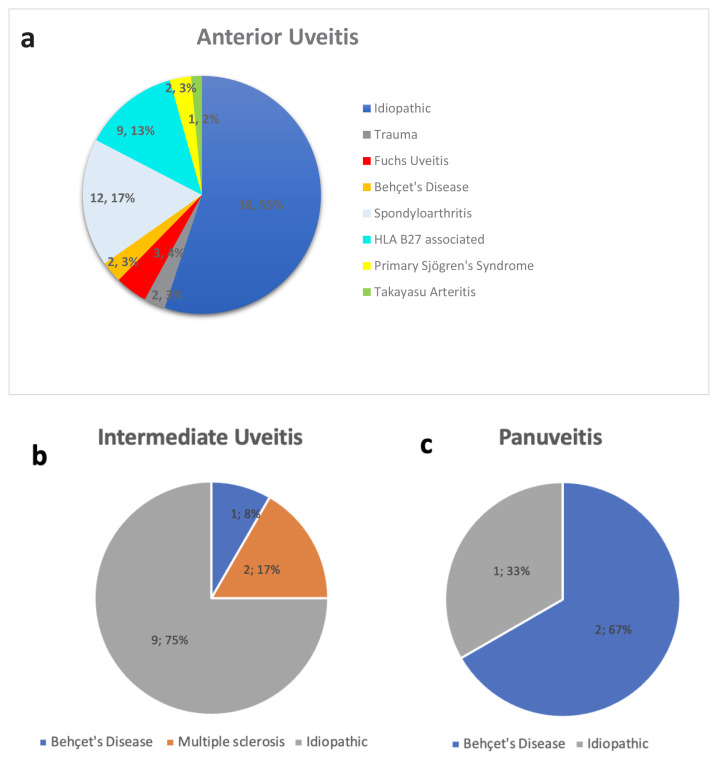
Etiological distribution of noninfectious uveitis by anatomical subtype: anterior (a), intermediate (b), and panuveitis (c).

Patients with infectious uveitis (five with AU, one with panuveitis, and one with IU) were excluded from the NIU subgroup analysis. The female-to-male ratio was 2:1 in panuveitis and IU, and 1.9:1 in AU. Idiopathic uveitis accounted for 55% of AU, 75% of IU, and 33% of panuveitis cases (Figures 2a and 2c). Among patients with SpA, 66% (n = 8) had ankylosing spondylitis and 33% (n = 4) had nonradiographic axial SpA. A breakdown of NIU etiologies by anatomical location is shown in Figure 2.

### 3.3. Rheumatologic causes of uveitis according to anatomic localization

**Figure 3:**
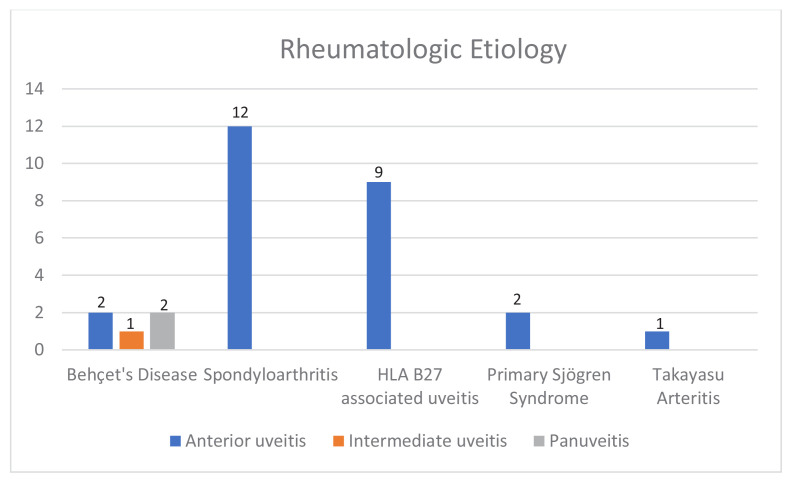
Rheumatologic causes of uveitis by anatomical subtype.

**Figure 4:**
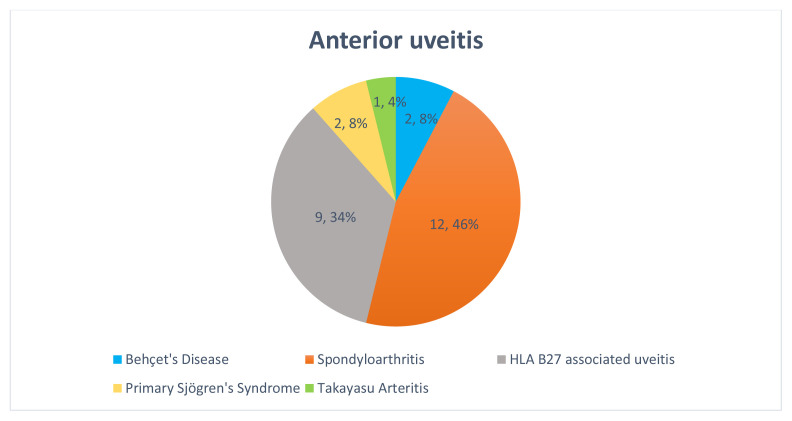
Distribution of anterior uveitis patients by rheumatologic diagnosis.

Rheumatologic causes were identified in 29 patients (31.9%), while 48 patients (52.7%) had idiopathic uveitis. Among patients with rheumatologic etiology, 26 (90%) had AU, two (7%) had panuveitis, and one (3%) had IU (Figure 3). Behçet’s disease was the underlying diagnosis in all cases of panuveitis and IU with rheumatologic etiology (Figure 3). Among AU cases with rheumatologic causes, 12 (46%) were diagnosed with SpA, nine (34%) with HLA-B27-associated uveitis, two (8%) with Behçet’s disease, two (8%) with pSS, and one (4%) with both TAK and HLA-B27 positivity (Figure 4).

### 3.4. Comparisons between patients with a rheumatologic diagnosis and the idiopathic group

Extraocular findings were significantly more common in patients with rheumatologic diagnoses. None of the patients with rheumatologic uveitis had granulomatous inflammation. Age, sex, uveitis type and laterality, treatment regimens, complications, comorbidities, and laboratory parameters (excluding HLA-B27) did not differ significantly between the two groups. Detailed comparisons between the groups are presented in Table 2.

### 3.5. Ocular complications in patients with rheumatologic etiology

Among uveitis patients with rheumatologic etiology, ocular complications were observed in five individuals (17.2%): four (80%) with AU and one (20%) with panuveitis. Hypertension and azathioprine use were significantly more frequent among those who developed complications. No significant differences in acute-phase reactants were observed between patients with and without complications. Table 3 presents detailed data comparing patients with and without complications.

**Figure 5:**
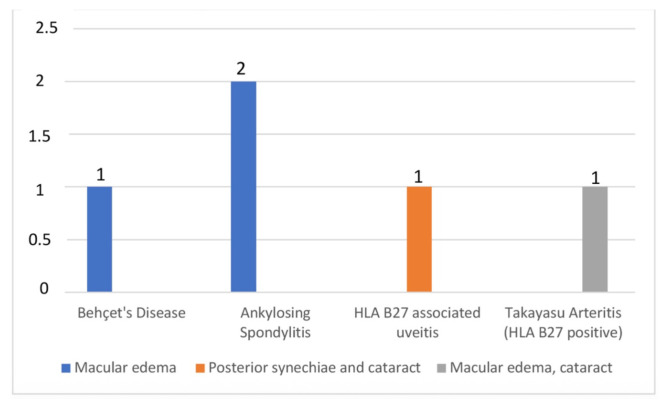
Ocular complications by rheumatologic diagnosis.

Macular edema was the most common ocular complication (13.8%), followed by cataract (6.9%), and posterior synechiae (3.4%). Macular edema occurred in patients with ankylosing spondylitis, Behçet’s disease, and TAK with HLA-B27 positivity (Figure 5). Ocular complications were observed in two of the 13 HLA-B27-positive patients (15.4%), while none of the 10 HLA-B27-negative patients experienced such complications. This difference was not statistically significant (Fisher’s exact test, p = 0.19).

## 4. Discussion

In this study, we analyzed demographic data, clinical features, laboratory tests, associated rheumatologic diseases, treatments, and complications of newly diagnosed uveitis patients referred to our rheumatology department over a 3-year period. A rheumatologic cause was identified in one-third of all uveitis patients, and ocular complications occurred in approximately one in five patients with newly diagnosed uveitis of rheumatologic etiology. Macular edema was the most common complication observed in the entire study population and in uveitis patients with rheumatologic etiology.

It is widely recognized that the incidence of NIU is higher in adult females than in adult males [1, 2, 9, 10]. Given the established fact that autoimmune disorders are more prevalent among the female population and that autoimmune uveitis is the primary cause of NIU, it is logical to hypothesize that adult female patients may be more susceptible to autoimmune uveitis. However, global data encompassing both infectious and noninfectious etiologies suggest a relatively equal sex distribution of uveitis overall [2]. Female predominance (65%) was observed in both the overall uveitis cohort and in NIU patients in our study. The female predominance in our uveitis cohort may be attributed to the lower proportion of infectious cases in the present study compared with population-based studies.

Regarding anatomical localization, the most common type was AU, observed in nearly 80% of patients, consistent with findings from large epidemiological studies [2, 21–23]. Approximately half of the patients had idiopathic uveitis, consistent with findings from Chinese and Italian population studies [7, 24]. Nearly one-third of the patients were diagnosed with a systemic autoimmune disease. In line with previous research, the most common rheumatic conditions were SpA and HLA-B27-positive uveitis [3]. Of the patients tested for HLA-B27, 56% were positive. Sentinel, a large multicenter prospective study, investigated SpA in 798 AU patients [25]. They included AU patients with known HLA-B27 status, more than one episode of AU, and no previous diagnosis of SpA. The percentage of HLA-B27 positivity was 60%, similar to that in our study. Juanola et al. and Pato et al. reported that half of the AU patients were found to have undiagnosed SpA [25, 26]. Fernández-Melón et al. concluded that uveitis was the first clinical sign of SpA in 41% of 394 patients [27]. In our cohort, 28.3% of AU patients had SpA or HLA-B27 positivity.

Uveitis is a rare manifestation of pSS; however, anterior, posterior, and panuveitis have all been documented in the literature [7, 28]. In our cohort, pSS accounted for 8% of the rheumatologic causes of AU, whereas previous studies have reported the prevalence of pSS among patients with AU of rheumatologic origin to be between 0.5% and 4% [3, 6, 7]. The pathogenesis of uveitis associated with pSS remains unclear, but it is currently believed to be related to autoimmune mechanisms [28–30]. Common immunological mechanisms—such as the involvement of Th1 lymphocytes in the early stages of disease, the expression of interleukin-17 (IL-17) and Th17 cells, the activation of inflammasomes and matrix metalloproteinases, and the infiltration of innate immune cells—support this view of the shared pathogenesis of uveitis and dry eye disease [31]. Ocular complications of pSS may include dry eye, corneal melting and perforation, scleritis, retinal vasculitis, optic neuritis, and uveitis [32]. Although uveitis and dry eye in pSS can share symptoms such as blurred vision, photophobia, and decreased visual acuity, their pain characteristics differ: dry eye typically presents with neuropathic pain, whereas uveitis is associated with a dull ocular pain that worsens with focusing. Furthermore, ocular redness appears as ciliary injection in uveitis, whereas it presents as diffuse hyperemia in dry eye syndrome [31]. Awareness of these clinical features, along with patient education, is essential for rheumatologists to ensure the early recognition and diagnosis of uveitis in individuals with pSS.

Takayasu arteritis is a rare cause of uveitis. According to a case-based systematic review and metaanalysis investigating ocular findings in TAK patients without other autoimmune diseases, uveitis was reported in five cases (four AU and one panuveitis) (4.1%), four of whom were children [33]. Only Becker et al. [34] did not report HLA-B27 status; the other four cases were negative for HLA-B27 [35–38]. The cooccurrence of TAK and SpA has been highlighted in numerous studies [39–42], and the pathogenesis of TAK appears to be mediated by the major histocompatibility complex (MHC) class I allele HLA-B*52, which is also implicated in SpA-related disorders [43]. Moreover, studies demonstrating the association between TAK and the SpA spectrum suggest that this overlap is unlikely to be incidental, supporting the notion that TAK may be classified within the emerging group of disorders known as “MHC class I-opathies” [44, 45]. In the study by Abacar et al., the prevalence of uveitis was reported as 1.8%, together with data from other studies in the literature investigating the frequency of SpA features in TAK patients [39]. Kwon et al. reported that the features of sacroiliitis in patients with TAK differed from those typically observed in SpA, noting a predominance of female patients and a notably low frequency of HLA-B27 positivity (14.3%) [42]. Moreover, Güzel Esen et al. reported that, among 14 patients diagnosed with TAK and SpA, one of the four who developed uveitis was HLA-B27 positive [41]. Our patient with TAK who developed AU did not fulfill the ASAS criteria for SpA but was monitored for the potential development of SpA.

Ocular involvement is present in approximately 70% of individuals with Behçet’s disease and usually manifests as recurrent, remitting uveitis [46]. Anterior or posterior uveitis, or more commonly panuveitis, is the typical form of uveitis affecting patients with Behçet’s disease. However, several cohort studies have also reported IU in patients with Behçet’s disease [47–51]. A multicenter study from Türkiye reported that Behçet’s disease accounts for 31.3% of AU, 41.7% of posterior uveitis, and 53.8% of panuveitis patients [52]. In our study, 50% of patients with panuveitis and 8% of those with AU had Behçet’s disease. Patients diagnosed in the past were not included in our cohort; this exclusion may explain the lower proportion of Behçet-related AU and panuveitis in our study compared to previous reports.

Although IU represents the least common anatomical subtype of uveitis globally (10%–15%) [2, 53], it was the second most frequent form in our cohort, accounting for 14% of patients. A minor female preponderance was observed in the majority of adult patient cohorts [51]. In our cohort, the female-to-male ratio was 1.6:1, which was consistent with the literature. Idiopathic uveitis was reported most frequently in IU compared with anterior, posterior, and panuveitis in most studies [51]. In our cohort, 75% of patients in the IU group were idiopathic, representing the highest rate of idiopathic cases among all uveitis types. From the standpoint of etiology, sarcoidosis, multiple sclerosis, and HLA-B27-associated diseases are the most commonly reported systemic conditions [51]. However, patients with HLA-B27-associated AU have a 10-fold increased risk of concomitant IU compared with patients without the HLA-B27 haplotype [54]. In our cohort, multiple sclerosis was the most commonly associated systemic condition in patients with IU. Additionally, juvenile idiopathic arthritis, Behçet’s disease, and granulomatosis with polyangiitis are less frequently reported systemic conditions associated with IU. Other causes include infections, drug associations, and malignancies [51].

Macular edema, glaucoma, cataract, and posterior synechiae were the most common complications observed in our cohort. Ocular complications were observed in 17% of patients with rheumatologic etiology. Concomitant hypertension and azathioprine requirements were significantly higher in rheumatology patients with complications. Although the difference in complication rates between HLA-B27-positive and HLA-B27-negative patients did not reach statistical significance, the absence of ocular complications in the HLA-B27-negative group may still be of clinical interest. This observation, while limited by the small sample size, raises the possibility of a protective association that warrants further investigation. Interestingly, a metaanalysis by D’Ambrosio et al. similarly reported no statistically significant difference in the overall rates of complications—such as posterior synechiae, cataract, and macular edema—between HLA-B27-positive and HLA-B27-negative patients, suggesting that the relationship between HLA-B27 status and ocular complications remains inconclusive and merits further study in larger populations [55]. These findings suggest that although HLA-B27 positivity is linked to distinctive inflammatory features, it may not be a determining factor for long-term structural complications, emphasizing the importance of timely recognition and management in both patient groups. Macular edema, which is the most common structural complication of uveitis and a major cause of central vision loss [56], was the most frequently observed complication in our cohort. Reported rates of macular edema ranged from 9%–28% in AU, 25%–70% in IU, 19%–34% in posterior uveitis, and 18%–66% in panuveitis [57]. Macular edema results from fluid accumulation in the central retina due to disruption of the blood-retinal barrier (BRB), a key feature shared by its underlying causes. Elevated levels of inflammatory cytokines and growth factors, such as vascular endothelial growth factor-A and tumor necrosis factor-α, contribute to BRB breakdown. This leads to an imbalance in osmotic and hydrostatic forces, ultimately impairing fluid homeostasis within the retina. Existing therapeutic approaches focus on modulating key pathways implicated in angiogenesis, inflammatory responses, and the disruption of the blood-retinal barrier [58]. As antiinflammatory treatments may influence the development of macular edema in previously diagnosed patients, only newly diagnosed patients presenting with uveitis were included in the study. Cataract and posterior synechiae are the other complications observed in patients with rheumatologic etiology. Although Behçet’s disease is known to be associated with severe ocular complications, ocular complications can also occur in SpA and HLA-B27-associated uveitis.

The lack of HLA-B27 data in about one-third of AU patients was a limitation of this study. Although some trends—such as the presence of hypertension or azathioprine use among patients with complications—were noted in our cohort, the low event rates limited our ability to perform robust statistical analyses. These preliminary observations should be considered hypothesis-generating and interpreted with caution, pending validation in larger, prospective studies. Additionally, the predominance of patients with previously diagnosed Behçet’s disease and the limited number of newly diagnosed cases have restricted our ability to draw conclusions about this important condition. The exclusion of previously diagnosed patients from the study allowed us to focus on newly diagnosed patients and their complications.

Our findings underscore that even among patients with no prior rheumatologic diagnosis, a significant proportion—nearly one-third—were ultimately found to have an underlying systemic autoimmune disease. This highlights the importance of routine etiologic investigation in cases of new-onset uveitis, which may be the initial presentation of a rheumatologic disorder. Moreover, ocular complications were observed in a notable subset of patients, regardless of the anatomical type, reinforcing the need for vigilant monitoring. These results support the integration of rheumatologic evaluation into the diagnostic workup of uveitis and emphasize the value of interdisciplinary care. Enhancing awareness among rheumatologists regarding early ocular symptoms and among ophthalmologists regarding systemic associations could improve patient outcomes through earlier diagnosis and coordinated management.

## 5. Clinical implications and future directions

This study underscores the risk of ocular complications in adult patients with uveitis presenting to rheumatology in Türkiye. Further large-scale prospective studies are warranted to elucidate the underlying risk factors associated with these complications.

## Figures and Tables

**Table 1:** Demographic, laboratory, systemic, treatment, and ocular complication characteristics of uveitis patients.

Characteristic	n = 91
Female sex n (%)	59 (64.8)
Age (year) (mean±SD)	45.7 ± 14.8
Uveitis laterality, n (%)	
Unilateral	46 (50.5)
Bilateral	45 (49.5)
Uveitis type, n (%)	
Anterior	74 (81.3)
Intermediate	13 (14.3)
Posterior	0 (0)
Panuveitis	4 (4.4)
Presence of granuloma, n (%)	
Nongranulomatous	71 (78)
Granulomatous	20 (22)
Uveitis chronicity, n (%)	
Acute	88 (96.7)
Chronic	3 (3.3)
Etiology, n (%)	
Idiopathic	48 (52.7)
Rheumatologic	29 (31.9)
Infectious	7 (7.7)
Trauma	2 (2.2)
Fuchs	3 (3.3)
Demyelinating disease	2 (2.2)
Noninfectious uveitis	84 (92)
Rheumatologic diagnosis, n (%)	
Nonrheumatologic	62 (68.1)
Spondyloarthritis	12 (13.2)
HLA-B27 associated uveitis	9 (9.9)
Behçet disease	5 (5.5)
Primary Sjögren	2 (2.2)
Takayasu Arteritis	1 (1.1)
Treatment, n (%)	
None	4 (4.4)
Topical	75 (82.4)
Systemic steroids	4 (4.4)
Azathioprine	5 (5.5)
Cyclosporine	1 (1.1)
Adalimumab	2 (2.2)
Antibiotics	1 (1.1)
Complications (n, %)	
None	72 (79.1)
Macular edema	12 (13.2)
Cataract	7 (7.7)
Glaucoma	6 (6.6)
Posterior synechiae	1 (1.1)
Extraocular findings, n (%)	
None	66 (71.4)
Musculoskeletal	15 (16.4)
Mucocutaneous	8 (8.8)
Neurologic	2 (2.2)
Vascular	1 (1.1)
Comorbidity, n (%)	
Hypertension	17 (18.7)
Diabetes Mellitus	9 (9.9)
Hyperlipidemia	9 (9.9)
Coronary Heart Disease	3 (3.3)
Others (hypothyroidism, depression, gout)	4 (4.4)
Laboratory, n (%)	
HLAB27 positivity^*^, n = 23	13 (56.5)
HLAB51 positivity^*^, n = 5	4 (80)
CRP (median (min–max))	2 (1–4)
ESR (median (min–max))	5 (4–10)
ANA positivity, n = 53	5 (9.4)
ANCA positivity, n = 30	1 (3.3)
ACE (mean ± SD)	43.7 ± 18.3

ACE: angiotensin-converting enzyme; ANA: antinuclear antibody; CRP: C-reactive protein; ESR: erythrocyte sedimentation rate.

**Table 2:** Comparison between rheumatologic and idiopathic uveitis cases.

Characteristic	Idiopathic, n = 48	Rheumatologic, n = 29	Total n = 77	p value
Sex				0.68^a^
Female sex	32 (66.7)	18 (62.1)	50 (64.9)	
Age (year)+	44.4 ± 16.2	46.3 ± 12.1	45.1 ± 14.7	0.59^b^
Uveitis laterality*				0.62^a^
Unilateral	22 (45.8)	15 (51.7)	37 (48.1)	
Bilateral	26 (54.2)	14 (48.3)	40 (51.9)	
Uveitis type*				0.10^a^
Panuveitis	1 (2.1)	2 (6.9)	3 (3.9)	
Intermediate uveitis	9 (18.8)	1 (3.4)	10 (13)	
Anterior uveitis	38 (79.2)	26 (89.7)	64 (83)	
Presence of granuloma*				**0.003** c
Nongranulomatous	36 (75)	29 (100)	65 (84.4)	
Granulomatous	12 (25)	0 (0)	12 (15.6)	
Therapy*				
None	2 (4.2)	1 (3.4)	3 (3.9)	>0.99^c^
Topical	39 (81.3)	26 (89.7)	65 (84.4)	0.52^c^
Systemic steroids	3 (6.3)	0 (0)	3 (3.9)	0.29^c^
Azathioprine	2 (4.2)	2 (6.9)	3 (3.9)	0.63^c^
Cyclosporine	1 (2.1)	0 (0)	1 (1.3)	>0.99^c^
Adalimumab	2 (4.2)	0 (0)	2 (2.6)	0.52^c^
Complications *				
None	38 (79.2)	24 (82.8)	62 (80.5)	0.70^a^
Macular edema	6 (12.5)	4 (13.8)	7 (9.1)	>0.99^c^
Glaucoma	4 (8.3)	1 (3.4)	5 (5.9)	0.65^c^
Cataract	2 (4.2)	2 (6.9)	4 (5.2)	0.63^c^
Posterior synechiae	0 (0)	1 (3.4)	1 (1.3)	0.38^c^
Extraocular findings*				**<0.001** a
None	45 (93.8)	8 (31)	53 (68.8)	
Musculoskeletal	2 (4.2)	13 (44.8)	15 (19.5)	
Mucocutaneous	1 (2.1)	6 (20.7)	7 (9.1)	
Neurologic	0 (0)	1 (3.4)	1 (1.3)	
Vascular	0 (0)	1 (3.4)	1 (1.3)	
Comorbidity*				
Hypertension	6 (12.5)	6 (20.7)	12 (15.6)	0.35^c^
Diabetes Mellitus	3 (6.3)	3 (10.3)	6 (7.8)	0.67^c^
Hyperlipidemia	3 (6.3)	3 (10.3)	6 (7.8)	0.67^c^
Coronary Heart Disease	1 (2.1)	1 (3.4)	2 (2.6)	>0.99^c^
Others (hypothyroidism, depression, gout)	2 (4.2)	0 (0)	2 (2.6)	0.52^c^
Laboratory				
HLAB27 positivity*, n = 23	0 (0)	13 (76.4)	13 (56.5)	**<0.001** c
HLAB51 positivity*, n = 5	3 (75)	1 (100)	4 (80)	0.12^c^
CRP+	1 (1–3.8)	3 (1–5)	2 (1–4)	0.31^d^
ESR+	5 (4–9.5)	7.5 (5–12.8)	5 (4–10)	0.19^d^
ANA positivity*, n = 46	2 (5.7)	2 (18.2)		0.24^c^
ANCA positivity*, n = 25	1 (4.8)	0 (0)		>0.99^c^

* n (%)

+ mean ± SD or median (Q1–Q3)

a chi-square test

b independent-samples t-test

c Fisher’s exact test

d Mann–Whitney U test

**Table 3:** Comparison of characteristics between patients with and without ocular complications.

	Patients without ocular complications n = 24	Patients with ocular complications n = 5	p value
Sex*			>0.99^a^
Female	15 (62.5)	3 (60)	
Male	9 (37.5)	2 (40)	
Age+	45.5 (37.3–52.3)	56 (43.5–69)	0.078^b^
Uveitis laterality*			0.17^a^
Unilateral	14 (58.3)	1 (20)	
Bilateral	10 (41.7)	4 (80)	
Uveitis type*			0.41^c^
Panuveitis	1 (4.2)	1 (20)	
Intermediate uveitis	1 (4.2)	0 (0)	
Anterior uveitis	22 (91.7)	4 (80)	
Rheumatologic diagnosis*			0.25^c^
Behçet’s disease	4 (16.7)	1 (20)	
Spondyloarthritis	10 (41.7)	2 (40)	
HLAB27 positivity	8 (33.3)	1 (20)	
Takayasu Arteritis	0 (0)	1 (20)	
Sjögren Syndrome	2 (8.3)	0 (0)	
Therapy*			**0.006** c
None	1 (4.2)	0 (0)	>0.99^a^
Azathioprine	0 (0)	2 (40)	**0.025** a
Topical	23 (95.8)	3 (60)	0.068^a^
Extraocular findings*			0.17^c^
None	8 (33.3)	1 (20)	
Musculoskeletal	11 (45.8)	2 (40)	
Mucocutaneous	5 (20.8)	1 (20)	
Neurologic	0 (0)	1 (20)	
Comorbidity*			
Hypertension	2 (8.3)	4 (80)	**0.003** a
Diabetes Mellitus	1 (4.2)	2 (40)	0.068^a^
Hyperlipidemia	1 (4.2)	2 (40)	0.068^a^
Coronary Heart Disease	1 (4.2)	0 (0)	>0.99^a^
Laboratory			
HLAB27 positivity*, n = 18	11 (68.8)	2 (100)	>0.99^a^
HLAB51 positivity*, n = 18	1 (6.3)	0 (0)	>0.99^a^
CRP+	3 (1–5)	4 (2–11)	0.35^b^
ESR+	7 (5–10)	13 (4–17.5)	0.73^b^
ANA positivity*, n=11	1 (11.1)	1 (50)	0.35^a^
ACE+	45 (32–58)	22 (22–22)	0.67^b^

ACE: angiotensin-converting enzyme; ANA: antinuclear antibody; CRP: C-reactive protein; ESR: erythrocyte sedimentation rate.

* n (%)

+ mean ± SD or median (Q1–Q3)

a Fisher’s exact test

b Mann–Whitney U test

c chi-square test
